# How Does Circadian Rhythm Impact Salt Sensitivity of Blood Pressure in Mice? A Study in Two Close C57Bl/6 Substrains

**DOI:** 10.1371/journal.pone.0153472

**Published:** 2016-04-18

**Authors:** Roy Combe, John Mudgett, Lahcen El Fertak, Marie-france Champy, Estelle Ayme-Dietrich, Benoit Petit-Demoulière, Tania Sorg, Yann Herault, Jeffrey B. Madwed, Laurent Monassier

**Affiliations:** 1 Institut Clinique de la Souris, Institut de Génétique et de Biologie Moléculaire, Université de Strasbourg, Illkirch, France; 2 Laboratoire de Neurobiologie et Pharmacologie Cardiovasculaire, Faculté de Médecine, Fédération de Médecine Translationnelle, Université et Centre Hospitalier Universitaire de Strasbourg, France; 3 Merck Research Laboratories, Kenilworth, New Jersey, United States of America; George Washington University School of Medicine and Health Sciences, UNITED STATES

## Abstract

**Background:**

Mouse transgenesis has provided the unique opportunity to investigate mechanisms underlying sodium kidney reabsorption as well as end organ damage. However, understanding mouse background and the experimental conditions effects on phenotypic readouts of engineered mouse lines such as blood pressure presents a challenge. Despite the ability to generate high sodium and chloride plasma levels during high-salt diet, observed changes in blood pressure are not consistent between wild-type background strains and studies.

**Methods:**

The present work was designed in an attempt to determine guidelines in the field of salt-induced hypertension by recording continuously blood pressure by telemetry in mice submitted to different sodium and potassium loaded diets and changing experimental conditions in both C57BL/6N and C57BL/6J mice strain (Normal salt *vs*. Low salt *vs*. High-salt/normal potassium *vs*. High salt/low potassium, standard *vs*. modified light cycle, Non-invasive tail cuff blood pressure *vs*. telemetry).

**Results:**

In this study, we have shown that, despite a strong blood pressure (BP) basal difference between C57BL/6N and C57BL/6J mice, High salt/normal potassium diet increases BP and heart rate during the active phase only (dark period) in the same extent in both strains. On the other hand, while potassium level has no effect on salt-induced hypertension in C57BL/6N mice, high-salt/low potassium diet amplifies the effect of the high-salt challenge only in C57BL/6J mice. Indeed, in this condition, salt-induced hypertension can also be detected during light period even though this BP increase is lower compared to the one occurring during the dark period. Finally, from a methodological perspective, light cycle inversion has no effect on this circadian BP phenotype and tail-cuff method is less sensitive than telemetry to detect BP phenotypes due to salt challenges.

**Conclusions:**

Therefore, to carry investigations on salt-induced hypertension in mice, chronic telemetry and studies in the active phase are essential prerequisites.

## Introduction

High dietary salt intake has long been recognized as a risk factor for hypertension and stroke and a global reduction of sodium intake could contribute to a reduction of blood pressure (BP) in the general population [1]. Mouse transgenesis has provided a unique opportunity to address the mechanisms underlying sodium kidney reabsorption, as well as end organ damage. Despite high plasma sodium and chloride concentrations observed during high-salt (HS) regimen, changes in systemic BP are not consistent among wild-type (WT) control strains. A previous work analyzing the contribution of aldosterone synthase in the sensitivity of BP to salt, a high dietary salt regimen markedly increased water intake and urinary volume in parallel to a blunted renin and aldosterone secretion in 129/SvEv animals without any effect on the BP [2]. Similarly, in other transgenic mouse studies the salt challenge alone did not modify BP parameters [3–6]. Another published work identified some BP effects of HS intake. In C57BL/6J mice monitored with telemetry, a 3-week long HS diet induced a 7.4 mmHg increase in nocturnal systolic BP [7]. The diurnal BP was not affected. In a mixed background measured the same manner, a 7 day-long salt diet dose dependently increased the 24 hours average BP by around 15 mmHg [8]. Therefore, considering all these studies, to investigate the ability of a gene-targeted mutation to trigger hypertension associated with a HS intake seems easier than to study the role of a gene in the prevention and/or reversion of a hypertensive phenotype in the same conditions, since we cannot be sure that control animals will demonstrate reproducible responses.

Numerous studies addressed the question of this variability of BP response to salt intake in mice and different hypotheses can be considered. The first one is related to the genetic background of the animals [9]. 129/Sv mice exhibited higher BP than C57BL/6 under HS alone, and the hypertensive response was about 2.5 times greater in this strain when DOCA was added. Interestingly, female mice having 0, 1 or 2 copies of the angiotensin-converting enzyme (ACE) gene demonstrated a copy-dependent relationship between ACE gene expression and the response to high-salt diet [10]. This result indicates that polymorphisms affecting the ACE gene expression level could also explain the different BP response to salt in current mice strains. HS challenge can also uncover a BP phenotype in case of reduction of the nephron number [11]. In accordance with these results, when the angiotensinogen and renin genotypes were modified, a BP response to salt was mainly observed when the challenge was associated with nephrectomy and/or DOCA [12,13]. The mechanisms by which HS diet increases BP are not fully understood. An interesting hypothesis suggests that salt needs to cross the blood brain barrier (BBB) to activate a central ouabain Na, K-ATPase [14]. Therefore, salt plasma concentration would need to be high enough to cross the BBB and trigger hypertension through an increase of the sympathetic nervous system activity. A reduction of the nephron mass or use of a drug that reduces salt excretion would therefore increase and stabilize this concentration at a level that facilitates its crossing to the brain. Finally, in a given experimental condition, a HS regimen can trigger hypertension in a circadian-dependent manner. In a work studying the contribution of the capsaicin receptor TRPV1 in HS diet-induced hypertension, a 8% salt in the diet increased significantly the BP of control animals only during the dark i.e. activity period [15].

Taking into account that the BP response to salt diets in mice both depends on the animal itself and the experimental conditions, we designed a protocol aiming to address the following questions: (1) In a standard strain (C57BL/6N), what are the effects of salt switches (from low-salt (LS) to normal-salt and HS diets)? (2) Can we quantify circadian variations and is there a “cycle effect” (standard light/dark cycle versus reversed cycle)? (3) Could small variations in the genetic background (C57BL/6J versus C57BL/6N) modify the BP response to salt? (4) Is there an effect of potassium load? and finally, (5) Is telemetry more sensitive than non-invasive tail cuff BP to detect salt effects in mice?

## Materials and Methods

### Ethics

All protocols were approved by the animal research ethics committee (Com’Eth) of the Mouse Clinical Institute in accordance with the French and European Communities Council Directive EU/63/2010.

### Animals

10 week-old C57BL/6N and C57BL/6J male mice were respectively purchased from Taconic (Germantown, NY) and The Jackson Laboratories (Bar Harbor, ME). Food and water were freely available throughout the experiments. Mice were housed individually in open cages placed in ventilated housing enclosures with a controlled light cycle.

### Experimental protocol

A diagram of the experimental protocol is provided in S1 Fig. Twelve C57BL/6N and twelve C57BL/6J male mice were synchronized to a standard light/dark schedule of 12h/12h with lights on at 7:00 a.m., while twenty C57BL/6N and twenty C57BL/6J male mice were placed in a modified light/dark schedule of 12h/12h with lights on at 11:00 p.m. Mice were acclimated at least 2 weeks prior to the first BP recording.

All animals were implanted with telemetric devices at the age of 13 weeks. Only data sets from mice characterized by a stable mean arterial pressure (MAP) and a pulse pressure (PP) ≥ 20mmHg were included for further analysis. The surgeries for transmitters implantation had a success rate of 100%. However, during the 10 weeks of the experimental protocol some mice were discarded because of unstable BP or low PP due to clotting in the catheter of the telemetric probe. It is noteworthy that once an animal has been discarded during the course of the study, the integrality of its BP recordings were discarded (from the beginning until the observation of the BP recording issue) but was kept for non-invasive BP measurement. At the end, a total of 17 C57BL/6N and 17 C57BL/6J remained in the modified light cycle cohort while 11 C57BL/6N and 12 C57BL/6J were kept for the standard light cycle group.

For the standard light cycle cohort, mice were challenged 2 weeks with a standard normal-salt diet (0.25% sodium, 0.4% potassium, Research diets Inc.) followed by a LS diet (0.01% sodium, 0.4% potassium, Research diets Inc.) for two weeks. After this period the two groups were separated, half being fed during two additional weeks with a HS diet (3% sodium, 0.4% potassium, Research diets Inc.), while the other half was fed with a HS low-potassium diet (3% sodium, 0.01% potassium, Research diets Inc.). Telemetric recordings were performed every weekend. At the end, blood was collected by retro-orbital puncture under 2% isoflurane anesthesia after 4 hours fasting and mice were euthanized with CO_2_.

For the modified light cycle cohort, the chronology and food references were the same but the different salt challenges were during 3 weeks instead of 2 weeks to include the Non Invasive Blood Pressure (NIBP) test.

Plasma ion measurements under normal-salt and LS diets were performed on a third and separate cohort of 18 C57BL/6N and 20 C57BL/6J male mice, housed by 3 in open cages and under a modified light/dark cycle.

### Telemetry

Mice (25–30g) were implanted with TA11PA-C10 (Datasciences Inc., USA) telemetric implants under pentobarbital anesthesia (12%, Ceva Santé Animale, France). The catheter was placed into the ascending aorta via the right carotid artery and the emitter subcutaneously in the right flank. Animals were then allowed for at least 1 week recovery before first baseline recordings. Heart rate (HR), systolic (SBP), diastolic (DBP) and mean arterial pressure (MBP = DBP+1/3(SBP-DBP)) were collected using the Dataquest ART system version 1.1. PP was defined as the difference between SBP and DBP. Data were sampled by averaging the last 15 seconds of each 15 minutes period and stored on a hard disc. These measurements were performed during two consecutive days, every weekend to avoid disturbances (presence of coworkers in the animal facility). Thus, every weekend, 3 nights and 2 days were analyzed. Outliers values were identified as values out of the interval mean ±2 SD. This makes possible to clean the data sheets from exaggerated BP values due to compression of the catheter during certain specifics neck movements. Values were then presented as 12h means of all day and nights periods and expressed as means ±SEM.

### Non Invasive Blood Pressure (NIBP)

SBP and HR were measured by photoplethysmography with a computerized tail-cuff system (BP-2000, Visitech Systems, Apex, NC) in conscious animals as previously described [16]. Measurements were made in the room used for telemetric recordings at fixed diurnal interval (3:00 p.m.– 4:00 p.m.). When measurements were performed in periods corresponding to the dark period, the procedure was performed under a 2–3 Lux red light as a unique source of light.

### Plasma ions

Blood analyses were performed as previously described [17].

### Renin/aldosterone

The renin and aldosterone assays were performed in Dr. Blindingmaier’s lab at the Ludwig-Maximilians University in Munich (Germany). Aldosterone was measured by a competitive time-resolved fluorescence immunoassay using a biotinylated aldosterone tracer, as described in detail in Manolpoulo *et al*. paper [18]. Renin was measured using a commercially available radioimmunoassay kit (Angiotensin-I RIA, DiaSorin, Dietzenbach, Germany).

## Results

### Effect of LS and HS diets on BP in C57BL/6N mice

**Fig 1:**
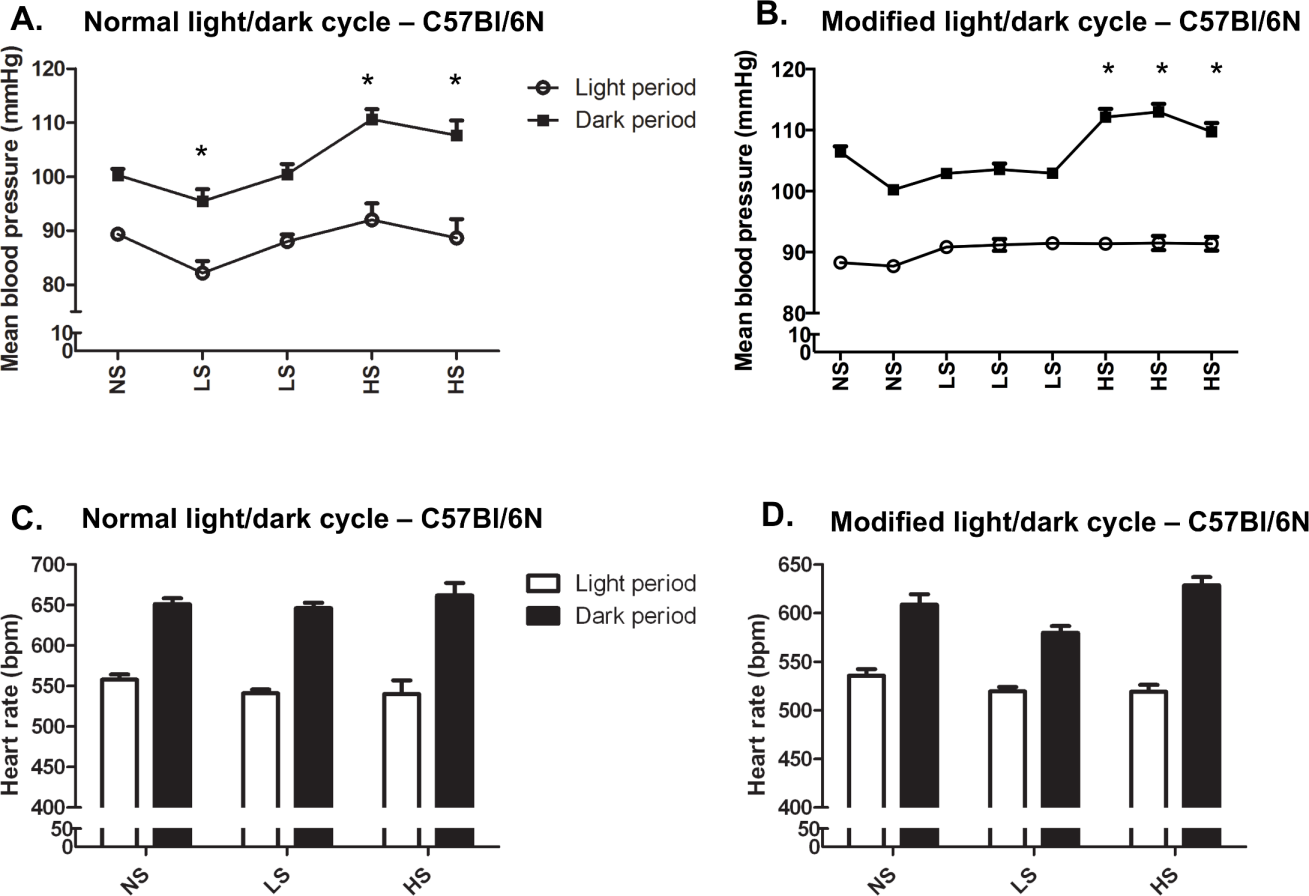
Effects of various salt challenges on mean blood pressure and heart rate in C57BL/6N male mice. Mice were monitored with telemetry and placed in a standard (A, C) or modified light/dark cycle (B, D). NS = normal salt diet (n = 12 and n = 17, for the standard and modified light cycle respectively), LS = low salt diet (n = 11 and n = 15, for the standard and modified light cycle respectively), HS = high Na^+^/normal K^+^ diet (n = 5 and n = 6, for the standard and modified light cycle respectively). One-way ANOVA per light phase followed by Tukey’s post-hoc test; *: p<0.05 compared to NS diet.

Under a standard light/dark cycle, two weeks of LS feeding induced a small and transient decrease in BP. This phenomenon was observed during the first week but was not maintained the second week (Fig 1A).

Mice were then placed under a HS diet (8% salt diet) for 2 weeks that could restrict the animals for normal feeding due to its taste. Hence, the palatability of this diet was assessed by measuring sodium excretion in a separated cohort as an indicator of the amount of Na^+^ ingested. Mice under HS diet showed an increase in sodium consumption as reflected by the 10 times increase in sodium urinary excretion compared to mice under a NS diet (Table 1) and the associated increase of water loss needed to dilute the urine to adjust osmotic pressure. This water loss was compensated by an increase in water intake. Taken together, these compensatory mechanisms led to maintenance of plasma sodium and potassium homeostasis.

**Table 1:** Sodium and potassium levels in urine and plasma in C57BL/6N.

Diet	n	Na^+^(U)	K^+^(U)	Na^+^(P)	K^+^(P)	Urinary volume (24h)	Water consumption (24h)	Body weight
		μmol/g/d	μmol/g/d	mmol/l	mmol/l	ml	ml	g
**NS**	**9**	9.3±0.5	16.3±0.7	146.4±0.5	4.7±0.1	1.4±0.1	5.4±0.4	30.9±0.9
**HS**	**10**	96.0±8.8*	6.4±0.4*	145.1±0.5	4.5±0.1	8.5±1.2*	13.9±1.7*	29.7±0.8

Urinary parameters (U) and plasma (P) ion measurement in a separate cohort of C57BL/6N male mice, under a standard light/dark cycle and normal salt diet (NS), or after 3 weeks of high Na+/normal K+ diet (HS). Data represented as mean +/- SEM. Student’s t-test

*: p<0.05 compared to NS diet.

On the other hand, HS diet had an effect on hemodynamic regulation. Indeed, compared to NS diet baseline values, HS diet significantly increased BP but only during the dark period. Indeed no difference in BP was observed between NS baseline values and HS diet values during the light period (Fig 1A). HR was never affected by the various salt challenges (Fig 1C).

### Effect of modified light/dark cycle on HS induced hypertension

The circadian variation of BP and HR was similar in C57BL/6N mice placed in a modified light/dark cycle compared to a standard light dark cycle (data not shown). Similarly, an increase in BP in response to the HS diet that was restricted to the dark period was observed (Fig 1B). However, in this cohort, we failed to reproduce the transient BP decrease that was observed in the first week of the LS regimen in the standard cycle group. Interestingly, a significant tachycardia was observed in parallel to the BP increase during the HS challenge (575±14bpm *vs*. 629±8bpm, p<0.05 Fig 1D). Despite a 26 bpm increase in the standard light dark cycle cohort (652±8bpm *vs*. 678±9bpm), this increase failed to achieve statistical significance.

No change in plasma ions was observed in C57BL/6N mice in response to the different salt regimen (Table 2). Efficiency of different salt challenges was assessed by measuring plasma renin activity and aldosterone concentration. These two parameters are known to be tightly regulated by salt intake. As expected, three weeks of LS feeding induced a marked increase in aldosterone concentration associated with a higher renin activity (Table 2). Conversely, HS diets induced a marked repression of plasma renin activity compared to normal salt diet, and aldosterone production was totally suppressed.

**Table 2:** Plasma ion, aldosterone and renin activity measurements in C57BL/6N and C57BL/6J male mice.

**Strain**	Diet	n	Na^+^	K^+^	Cl^-^	Mg^2+^	Aldosterone	Renin activity
			mmol/l	mmol/l	mmol/l	mmol/l	pg/ml	ng/ml/hr
**C57BL/6N**	**NS**	**9**	150.2±0.4	4.2±0.1	114.3±0.4	0.99±0.03	336±51	80±5
	**LS**	**9**	150.1±0.5	4.0±0.2	112.0±0.4*	0.87±0.02*	541±61*	177±33*
	**HS**	**8**	148.6±0.5	4.5±0.2	113.6±0.3	0.97±0.03	≤36*	48±5 *
	**HS/LK**	**8**	151.3±0.7	4.3±0.2	112.5±1	1.04±0.04	≤36*	34±6*
**C57BL/6J**	**NS**	**10**	151.5±0.4	4.5±0.1	117.1±0.7	1.00±0.03	272±16	76±16
	**LS**	**10**	150.0±0.4	4.1±0.1	114.1±0.7*	0.91±0.03	562±52*	174±21*
	**HS**	**9**	149.3±0.5*	4.5±0.2	114.5±0.4*	0.94±0.04	66±4*	38±5*
	**HS/LK**	**8**	150.5±0.6	3.3±0.3*	114.6±0.9*	1.01±0.03	75±4*	12±2*

Plasma ion measurements performed on a third and separate cohort of 18 C57BL/6N and 20 C57BL/6J male mice, under a modified light/dark cycle. NS = normal salt diet, LS = low salt diet, HS = high Na+/normal K+ diet and HS/LK = high Na+/low K+ diet. Data represented as mean +/- SEM. One-way ANOVA per strain followed by Tukey’s post-hoc test

*: p<0.05 compared to NS diet.

### Comparison of HS induced hypertension between C57BL/6N and C57BL/6J mice

**Fig 2:**
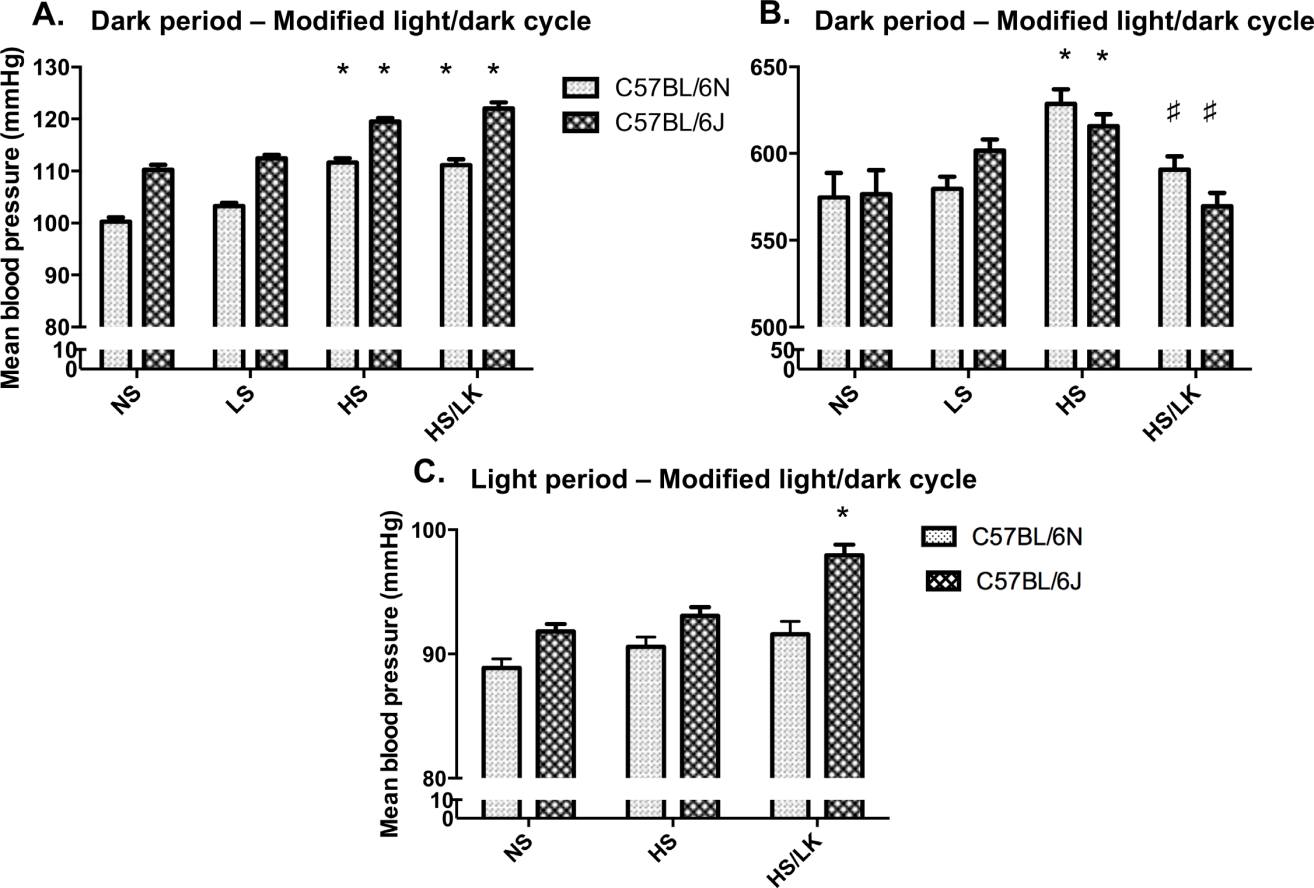
Comparison between C57BL/6N and C57BL/6J for their mean blood pressure and heart rate responses to various salt challenges. Mice were monitored with telemetry during the dark (A, B) and light (C) periods of a modified light/dark cycle. NS = normal salt diet (n = 17 and n = 17, for C57BL/6N and C57BL/6J respectively), LS = low salt diet (n = 15 and n = 17, for C57BL/6N and C57BL/6J respectively), HS = high Na^+^/normal K^+^ diet (n = 8 and n = 8, for C57BL/6N and C57BL/6J respectively) and HS/LK = high Na^+^/low K^+^ diet (n = 6 and n = 9, for C57BL/6N and C57BL/6J respectively). One-way ANOVA per light phase followed by Tukey’s post-hoc test; *: p<0.05 compared to NS diet; #: p<0.05 HS/LK compared to HS.

Whatever the applied salt regimen, C57BL/6J mice had a higher BP than C57BL/6N animals (+10±1mmHg; Fig 2A). In a modified light/dark cycle, LS diet modified neither the BP nor the HR in both substrains (Fig 2A and 2B). Similar to C57BL/6N, when fed a HS diet, C57BL/6J mice showed an increase in BP and HR in the dark period (+12mmHg in C57BL/6N and +10mmHg in C57BL/6J; Fig 2A) whereas BP was comparable between NS and HS fed mice during the light period (Fig 2C). In addition, a similar HR increase was also observed in the two substrains during the dark period (575±14bpm *vs*. 629±8bpm in C57BL/6N and 582±14mmHg vs. 615±8bpm in C57BL/6J; NS vs. HS; Fig 2B).

### Effect of potassium lowering on HS induced hypertension

**Fig 3:**
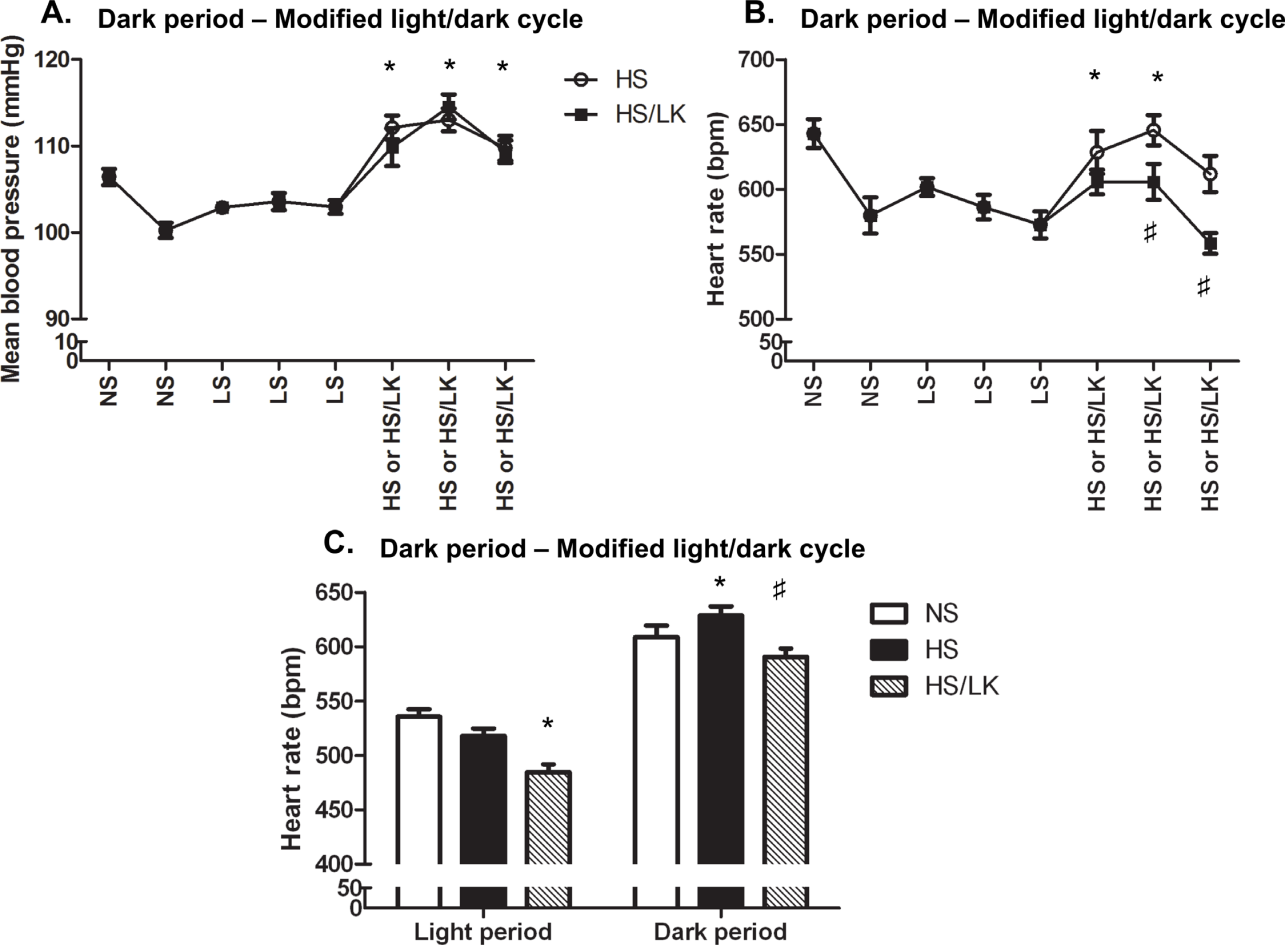
Effects of high-salt/normal potassium and high-salt/low potassium on mean blood pressure and heart rate in C57BL/6N mice. Mice were monitored with telemetry in the dark (A, B, C) and light (C) periods of a modified light/dark cycle. NS = normal salt diet (n = 17), LS = low salt diet (n = 15), HS = high Na^+^/normal K^+^ diet (n = 8) and HS/LK = high Na^+^/low K^+^ diet (n = 6). One-way ANOVA per light phase followed by Tukey’s post-hoc test; *: p<0.05 compared to NS diet; #: p<0.05 HS/LK compared to HS.

In C57BL/6N mice, when compared to normal potassium, the HS low potassium (HS/LK) intake did not affect the dark period BP increase that was superimposable in both groups (Fig 3A). Noteworthy, no statistically significant change in BP was observed during both HS/LK and HS diets during the light period (Fig 2C). Interestingly, HS/LK challenge prevented the HR augmentation observed in the HS group (Fig 3B & 3C). During the light period a significant bradycardia was observed in HS/LK group (518±11 bpm *vs*. 484±7 bpm, Fig 3C). The same pattern was observed when animals were placed in the standard light/dark cycle: during the dark period, BP increased by 12 mmHg and 11 mmHg for HS and HS/LK respectively when HR increased by 54 bpm in the HS group and by 15 bpm only in the HS/LK cohort (data not shown).

In C57BL/6J mice, HS/LK diet also induced a similar BP increase and prevented the dark period HR increase (Fig 2A & 2B). Moreover, in the light period, a 36 bpm reduction of the HR was observed when compared to the NS regimen (data not shown).

However, unlike C57BL/6N mice, C57BL/6J mice exhibited a slight but significant increase of BP during light period in response to HS/LK diet (92±1mmHg vs. 98±1mmHg; Fig 2C). Nevertheless, this increase was lower in extent than the one observed during the dark period (110±1mmHg vs. 122±1mmHg; NS vs. HS/LK). In the standard light/dark cycle, similar results were obtained (data not shown). Finally, in mice fed with a HS/LK diet, a decrease in plasma K+ concentration was observed in C57BL6/J mice only (Table 2).

### Effect of HS and HS/LK diets on circadian blood pressure variations

**Fig 4:**
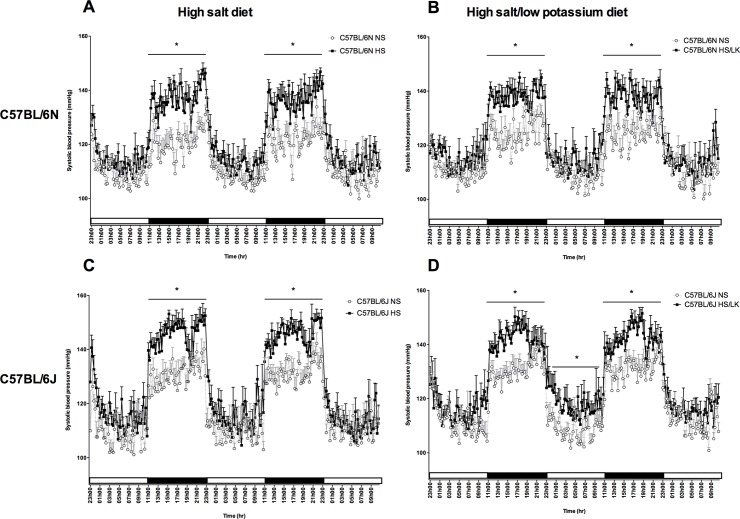
Effect of high salt diets on the circadian blood pressure variations in C57BL/6N and C57BL/6J mice under a reverse light/dark cycle. 2.5 days continuous telemetric recordings of systolic blood pressure after 2 weeks of NS, HS or HS/LK diet challenge. A) n = 8 per group B) n = 6 per group C) n = 8 per group D) n = 9 per group. Two-way ANOVA followed by Sidak’s post-hoc test *: p<0.05 for interaction.

The effects of the different high salt diets on BP were not due to a disturbance of the circadian rhythm neither in mice under a normal light/dark cycle nor under a modified light/dark cycle (Fig 4). Both C57BL6/N and C57BL6/J mice exhibited a decrease in BP during resting period i.e. when the light was turned on at 11:00 pm and an increase in BP during the active period i.e. when the light was turned off at 11:00 am. The shifts in BP were consistent from day to day and occurred at the same time as the light cycle showing a good adaptation of mice to experimental changes of the light/dark cycle. The circadian BP variation was similar in mice fed a NS diet compared to mice fed with HS or HS/LK diets in both strains. Therefore, a shift in the circadian BP rhythm cannot explain the dark-period restricted hypertension phenotype induced by the different high-salt diets.

### Comparison of NIBP and telemetry for assessment of HS-induced hypertension

**Fig 5:**
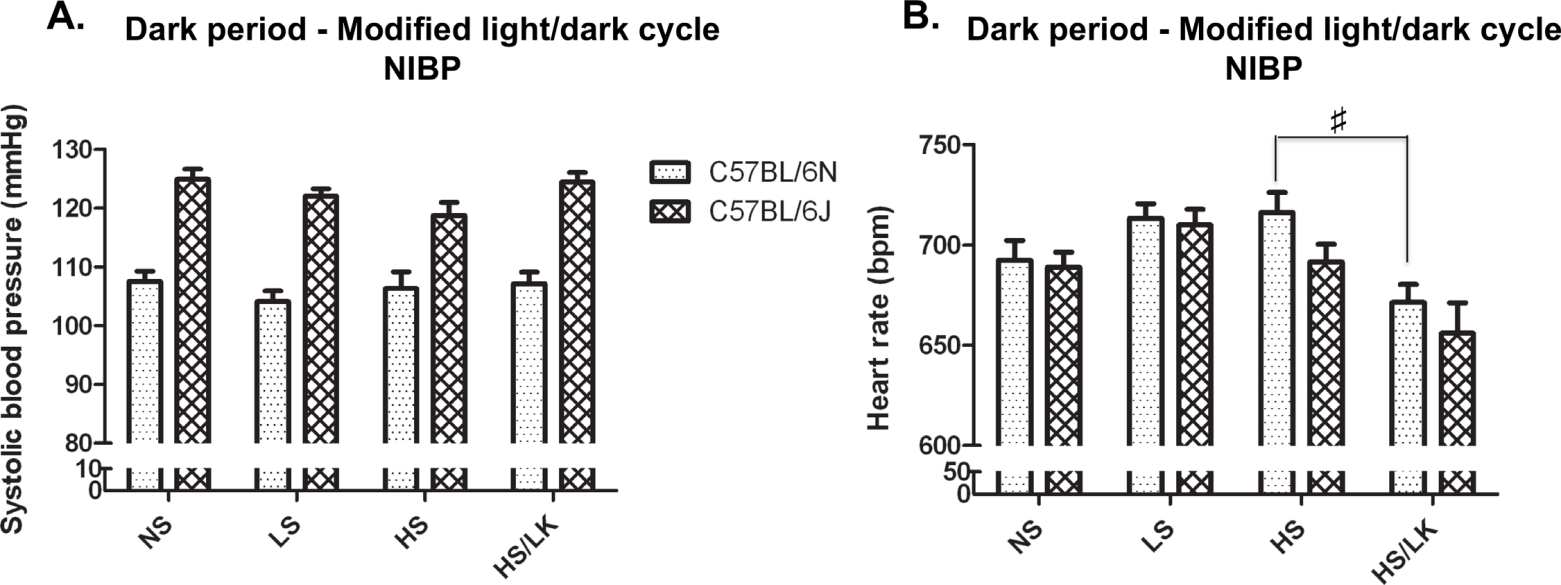
Comparison between C57BL/6N and C57BL/6J for their systolic blood pressure and heart rate responses to various salt challenges measured by NIBP. Measurements were made during the dark periods of a modified light/dark cycle. NS = normal salt diet (n = 20 and n = 20, for C57BL/6N and C57BL/6J respectively), LS = low salt diet (n = 20 and n = 20, for C57BL/6N and C57BL/6J respectively), HS = high Na^+^/normal K^+^ diet (n = 9 and n = 10, for C57BL/6N and C57BL/6J respectively) and HS/LK = high Na^+^/low K^+^ diet (n = 9 and n = 10, for C57BL/6N and C57BL/6J respectively). One-way ANOVA per light phase followed by Tukey Kramer’s post-hoc test; #: p<0.05 HS/LK compared to HS.

NIBP confirmed the higher baseline BP in C57BL/6J compared to C57BL/6N that was detected with telemetry. Despite the measurements performed during the dark period, no BP change in response to different salt diets was observed (Fig 5A). Nevertheless, the NIBP method successfully reproduced the lower HR showed by mice fed with HS/LK compared to the HS diet (Fig 5B). However, the HR increase observed with telemetry for the HS fed mice failed to be reproduced by NIBP.

## Discussion

In the present study, we have shown that HS diet increases BP and HR in mice, but the increase is observed during active period only. This phenotype is not explained by an increase in physical activity (data not shown) or a shift in circadian rhythm induced by the HS diet. Indeed there were no major difference neither in total ambulatory activity recorded by the telemetry system nor in the analysis of the full rhythm pattern over a 72 hours period between NS and HS fed mice. No difference in BP standard deviation was noted between NS and HS groups (S2 Fig) suggesting that the BP effect is independent from physical activity *per se*. It is also noteworthy that no stereotypic behaviors were noticed during the regular clinical observations.

The results of this study stressed the effect of circadian rhythm on the development of HS-induced hypertension. As the autonomic nervous system is an important player that mediates both circadian rhythm and BP, an abnormal modulation of this pathway under HS diet could explain this phenotype restricted to the dark period. Moreover, substantial findings indicate that the sympathetic nervous system may be involved in salt-induced hypertension in humans [19,20] and animals [21–23]. Stocker *et al*. suggested that the central nervous system integrates a variety of inputs together with ongoing activity to generate a level of renal sympathetic nerve activity (RSNA). Dietary salt would alter the excitability of these central networks (like the rostral ventrolateral medulla, the primary regulator of the sympathetic nervous system and notably involved in the baroreflex) to amplify a given input to produce a much greater level of RSNA [24] leading to increase of water and ions retention and associated BP raise. The results of our study support this hypothesis where high-salt intake potentiates the nocturnal increase in BP in response to sympathetic outflows during the active phase of the mice. Sympathetic nerves targeting kidneys are well known to be at the center of long-term BP control and such increase in RSNA was previously described in several models of salt-sensitive hypertension [25–28]. Nevertheless, some conflicting data emphasized the lack of central mechanisms in resting vasomotor tone due to sympathetic nervous system (SNS) in salt-induced hypertension. Chen *et al*. did not show any contribution of the SNS in the control of vasomotor tone during HS challenge. In their conditions, no BP reduction was obtained by prazosin, an alpha-1 adrenergic antagonist [29]. Similarly, the work by McBryde *et al*. in rabbits does not support a SNS sensitizing effect of HS diet *per se* as they showed that HS diet did not influence RSNA or BP [26,30]. However this discrepancy likely reflects the various methodological approaches and factors that contribute to this relationship of SNA and BP. Indeed, these studies where conducted in the daytime without further stimulation of the SNS.

Another explanation for this dark period-restricted BP phenotype is the level of sodium in the cerebrospinal fluid, which is a critical factor to the BP response due to dietary sodium as suggested by some studies [31]. Therefore, salt plasma concentration would need to be high enough to cross the BBB and trigger hypertension. This would be the case only when mice eat and drink during their active i.e. night period. Such plasma peaks would not be detected after the 4 hours fasting prior to blood collection used in the present study or during the day phase.

C57BL/6N and C57BL/6J exhibited a 10 mmHg difference in BP which is in accordance with previously published data [32]. Despite this basal BP difference, a similar BP response to HS was observed in these two strains. However HS/LK diet amplified the effect of the HS challenge only in C57BL/6J mice during the light period. The deleterious effect of the combination between HS and low potassium on BP has been described elsewhere [32]. Moreover, differences in potassium and chloride homeostasis between the two strains are suggested by the hypokalaemia and hypochloridaemia observed in C57BL/6J only. This result emphasizes on the differences between the two strains that has been recently published by the Eumodic consortium [33]. Moreover, studies on the sodium/water balance as well as assessment of the renin-angiotensin system activity in different mice strains revealed great differences in renal physiology among strains [34]. Thus, further investigations will be necessary to clarify differences in potassium handling between the two strains of the present study.

Finally, considering that tail-cuff photoplethysmography (NIBP) and telemetry are both reference methods used in a number of scientific papers on the effect of high-salt diet on BP, we decided to compare them in terms of sensitivity for salt studies. BP measurements by NIBP successfully identified the lower HR in mice fed the HS/LK compared to the HS/NK diet and the difference in resting BP values between C57BL/6J and C57BL/6N. However, no increase in SBP induced by the HS challenge was detected by NIBP method even if the measurements were performed during the dark period. This is in accordance with previous literature data where no BP increase following a HS diet *per se* was observed using NIBP in WT mice [2,35–38], but in contrast to the hypertensive phenotype identified with telemetry. Several hypotheses could explain this discrepancy. Telemetry detects BP in the ascending aorta, a conductance vessel, while NIBP evaluates the pressure in the tail artery, a resistance vessel with high vasomotion capabilities. Indeed, it was described that sympathetic nerves innervating different vessels are differentially regulated [39,40]. The difference could also come from the higher stress response to NIBP due to contention, heating and interactions with the technician, as reflected by the higher HR obtained with this method. This could in turn diminish the difference between mice under NS and HS diets by increasing NS BP and HR values.

## Conclusions

Our study raises the question of the true absence of BP phenotype in non salt sensitive mice usually described in the literature and suggest that all experiments assessing the effect of salt on BP should: 1) use chronic aorta catheterization to assess BP, and 2) be carried out in the active phase of the animals i.e. during the nocturnal period. Therefore for practical reasons we have shown that these nocturnal animals can be placed under a reversed dark/light cycle without affecting their response to the HS diet. Finally, HS/LK diet amplifies the effect of the HS challenge in C57BL/6J only. Thus, while C57BL6/N background is appropriate to assess the effect of sodium on BP, one should consider that the response to HS/low potassium on this genetic background might not be adequate for the evaluation of translational comparisons to other species and humans. These accurate experimental conditions are an essential prerequisite to the experimental investigation of salt-induced hypertension in mice.

## Supporting Information

S1 Fig Experimental design. (PDF)

S2 Fig 24 hours continuous blood pressure recording in C57Bl6/J mice under a modified light/dark cycle. (PDF)
