# Supplementary figures and images for: How Does Circadian Rhythm Impact Salt Sensitivity of Blood Pressure in Mice? A Study in Two Close C57Bl/6 Substrains
Source: PLoS One. 2016 Apr 18;11(4):e0153472. doi: 10.1371/journal.pone.0153472 (PMC4835052; doi:10.1371/journal.pone.0153472)

**A. Standard Light/Dark cycle cohorts**

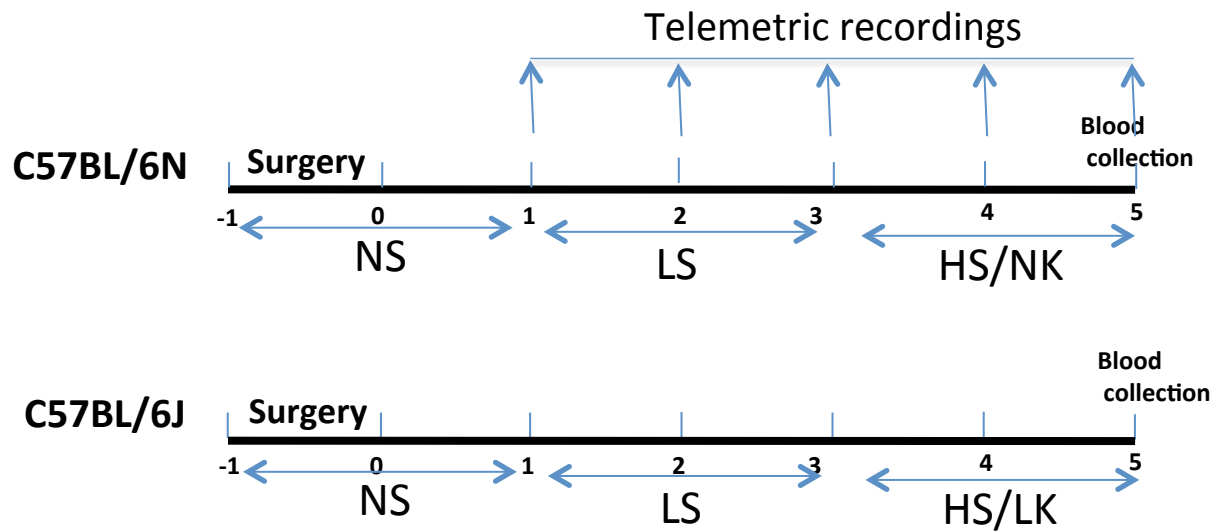

**B. Modified Light/Dark cycle cohorts**

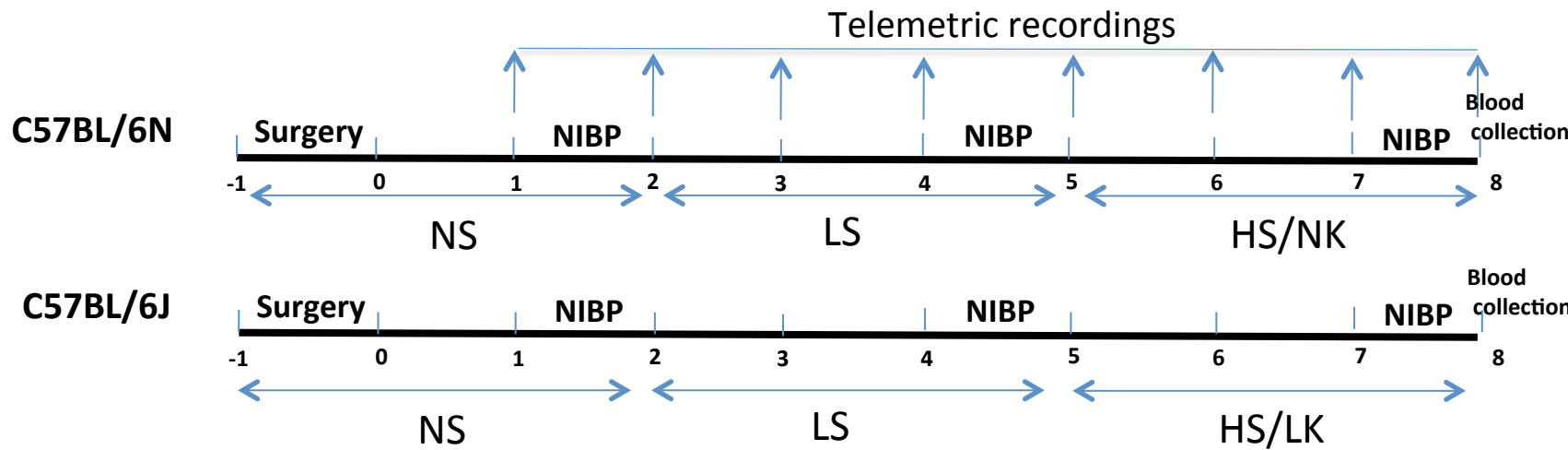

**C. Light/Dark cycle**

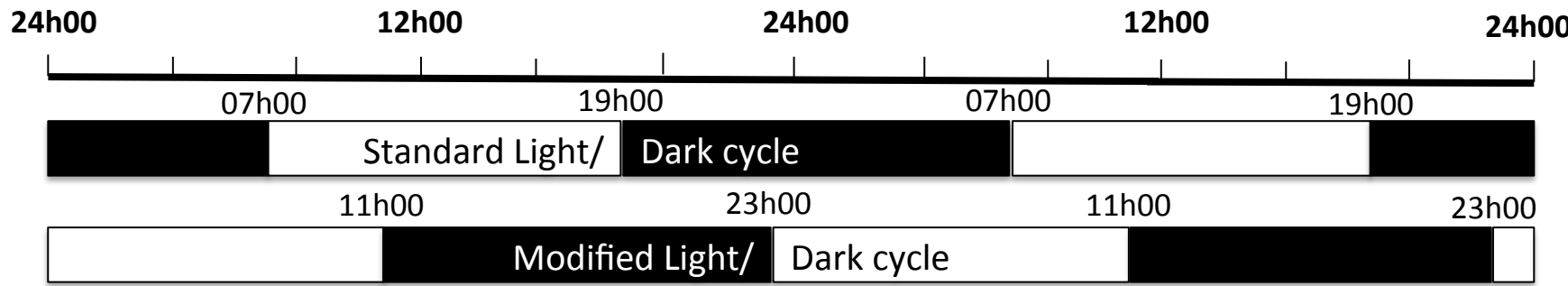

Supplement: S1 Fig — (PDF) [file pone.0153472.s001.pdf]
