# Supplementary material for: How Does Circadian Rhythm Impact Salt Sensitivity of Blood Pressure in Mice? A Study in Two Close C57Bl/6 Substrains
Source: PLoS One. 2016 Apr 18;11(4):e0153472. doi: 10.1371/journal.pone.0153472 (PMC4835052; doi:10.1371/journal.pone.0153472)
Supplement: S2 Fig — (PDF) [file pone.0153472.s002.pdf]

S2 fig. A : Tracings example of a continuous blood pressure recording in C57Bl6/J mice during 12 hours of the dark period. Dark lines represent mice under normal salt diet and colored lines represent mice under High salt/normal potassium diet.

A.

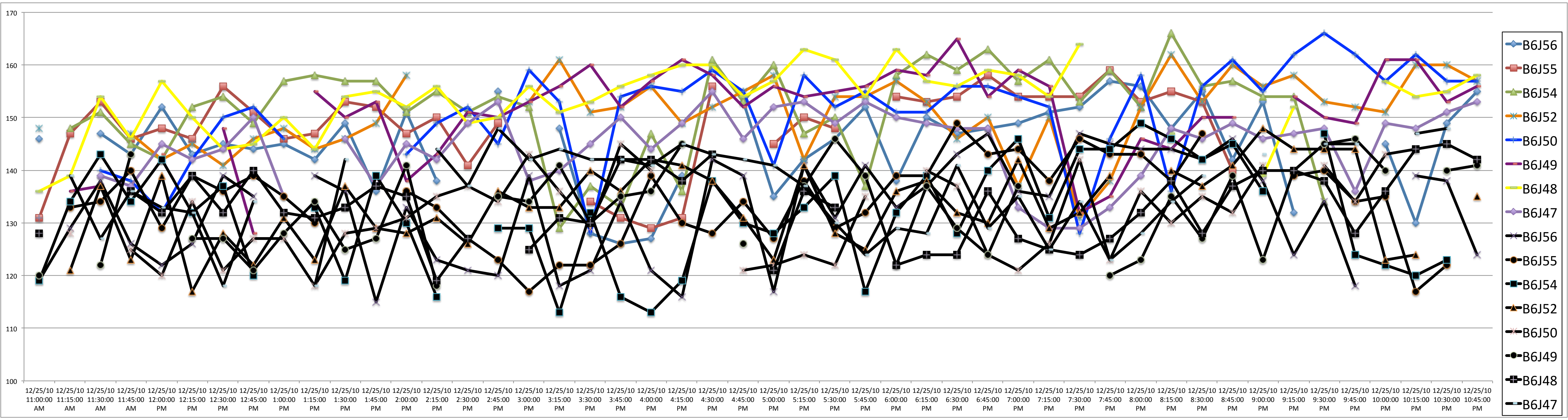

B.

	Standard deviation	B6J56	B6J55	B6J54	B6J52	B6J50	B6J49	B6J48	B6J47	Mean
Dark period	Normal salt	9.7	7.9	10.3	7.5	7.9	8.4	6.9	8.2	* 8.3
	High salt	8.2	8.1	8.7	6.8	9.4	8.4	6.5	6.6	* 7.9
Light period	Normal salt	6.4	4.9	5.3	8.0	8.9	6.4	7.0	6.7	6.7
	High salt	5.8	6.7	5.9	6.6	7.4	5.2	6.3	5.6	6.2

S2 fig. B : Standard deviation table of a continuous blood pressure recording in C57Bl6/J mice during 24 hours.. Standard deviation comparison were made using Bartlett's test (* $p<0.05$, Light vs. Dark period)
